# Multi-omics integration identifies ganoderic acid A as a TNFα inhibitor for treating sepsis-related liver injury

**DOI:** 10.3389/fphar.2026.1754172

**Published:** 2026-01-26

**Authors:** Hong Hu, Zike Chen, Jinlu Han, Mengyan Chen, Yun Song, De Zhao, Chen Wang, Min Shi

**Affiliations:** 1 Department of Gastroenterology, Tongren Hospital, Shanghai Jiao Tong University School of Medicine, Shanghai, China; 2 Hongqiao International Institute of Medicine, Tongren Hospital, Shanghai Jiao Tong University School of Medicine, Shanghai, China

**Keywords:** ganoderic acid A, inflammation, moleculardocking, network pharmacology, sepsis-related liver injury

## Abstract

Ganoderic acid A (GAA), a major bioactive triterpenoid from Ganoderma lucidum, is known for its anti-inflammatory effects; however, its precise molecular targets in sepsis-related liver injury (SRLI) remain unclear. Integrating network pharmacology and transcriptomic analysis, we identified Tumor Necrosis Factor-alpha (TNFα) as a primary candidate target. Subsequent biophysical validation using surface plasmon resonance (SPR) and molecular dynamics (MD) simulations confirmed that GAA directly binds to TNFα. Functionally, this interaction inhibits the TNFα/NF-κB signaling axis, thereby suppressing macrophage M1 polarization and ameliorating liver injury *in vitro* and *in vivo*. This study identifies TNFα as a primary candidate target of GAA, providing a mechanistic basis for its hepatoprotective effects and therapeutic potential.

## Introduction

Sepsis, a dysregulated host response to infection, remains one of the leading cause of mortality in intensive care units worldwide. Its progression is frequently complicated by multiple organ dysfunction, with sepsis-related liver injury (SRLI) being one of the most prevalent and detrimental, significantly exacerbating patient prognosis (Gu et al., 2024; Liang et al., 2022). The pathogenesis of SRLI is intricately linked to uncontrolled inflammatory responses, where macrophage-driven inflammation and the pivotal tumor necrosis factor-alpha (TNFα) signaling pathway play a critical role in driving hepatic damage (He et al., 2024). Currently, no targeted therapies have been approved, highlighting an urgent need for effective drugs.

In the search for novel therapeutic agents, natural products offer a rich reservoir of chemical diversity. The medicinal mushroom *Ganoderma lucidum*, listed simultaneously in the Chinese “Medicine–Food Homology” catalogue and in the European Novel-Food inventory, is renowned for its hepatoprotective and immunomodulatory properties (Li and Wang, 2006; Liu et al., 2020). These benefits are largely attributed to its unique triterpenoids, particularly Ganoderic acid A (GAA), which has demonstrated efficacy in various inflammatory and metabolic disease models (Cheng et al., 2013; Liu et al., 2020). However, while the bioactivities of GAA are well-documented, its precise molecular targets and the mechanism by which it exerts its effects in SRLI are unknown. Unclear mechanism limits its development as a targeted therapeutic or functional food ingredient.

Network pharmacology, a systems biology approach, provides a powerful tool for predicting the interactions between natural products and disease targets (Zhang et al., 2023; Zhao et al., 2023). We hypothesized that an integrative strategy, combining computational prediction with experimental validation, could effectively identify the key target and elucidate the mechanism of GAA in SRLI.

By integrating network pharmacology and liver transcriptomics in an SRLI mouse model, we proposed TNFα as a potential candidate target of GAA. This hypothesis was then evaluated through a combination of computational molecular docking, surface plasmon resonance (SPR), and molecular dynamics (MD) simulations to characterize the potential direct binding. Furthermore, we investigated whether this interaction might functionally inhibit the TNFα/NF-κB signaling axis, and whether this modulation could translate to suppressed pro-inflammatory macrophage polarization and attenuated liver injury *in vitro* and *in vivo*.

## Materials and methods

### Reagents and chemicals

The reagents and chemicals used in this study are listed in Supplementary Table S4.

### Cell model and drug treatment

The murine macrophage line RAW264.7 was pretreated with GAA (20 or 40 μM) for 1 h, followed by induction of inflammation with TNFα (50 ng/mL) for 12 h. GAA was prepared in dimethyl sulfoxide (DMSO), with a vehicle control containing an equivalent DMSO concentration (<0.1%). All cultures were maintained at 37 °C in a 5% CO_2_ atmosphere.

### Animal models and drug administration

An SRLI model was generated in male C57BL/6 mice (8 weeks old; n = 5 mice per group) as reported (Liu et al., 2025). GAA (20 or 40 mg/kg) or vehicle was administered daily by oral gavage for 3 days prior to lipopolysaccharide (LPS) challenge (10 mg/kg, i.p.). Mice were monitored for 10 h post-LPS injection before tissue collection under sodium pentobarbital anesthesia. The TNFα antibody group was administered 15 mg/kg anti-TNFα-antibody intraperitoneally along with the LPS injection. All animal studies were conducted per the approved protocol (Shanghai Tongren Hospital A2025-041-01).

### Network pharmacology

ETCM (http://www.tcmip.cn/ETCM), ChEMBL (https://www.ebi.ac.uk/chembl/) and SwissTargetPrediction (http://swisstargetprediction.ch/) were used to predict GAA targets. Using the terms “sepsis-related liver injury” and “septic acute liver injury,” therapeutic targets associated with SRLI were obtained from GeneCards (https://www.genecards.org/) (relevance score ≥10) and OMIM (https://www.omim.org/). To identify the key genes, we first converted the intersecting targets of GAA and SRLI into official gene symbols. We then constructed a protein-protein interaction (PPI) network based on these targets using a minimum interaction score of 0.4 (medium confidence). Subsequently, we conducted functional enrichment analysis on the common targets to systematically explore the core signaling pathways involved in GAA’s treatment of SRLI.

### Bioinformatics analysis of transcriptomic data

Bioinformatics Analysis of Transcriptomic Data The gene expression dataset GSE217695 was obtained from the Gene Expression Omnibus (GEO) database. This dataset includes liver tissue transcriptomic profiles from wild-type (WT) mice and mice with LPS-induced SRLI. Raw gene count matrices were obtained from the GEO dataset and pre-processed using the TMM (Trimmed Mean of M-values) method implemented in the edgeR package to correct for differences in sequencing depth and library size. Subsequently, the voom function in the limma package was applied to transform count data into log2-counts per million (logCPM) and estimate the mean-variance relationship for precision weighting. Differential expression analysis was then performed using linear models in limma. Genes with an absolute log2 fold change (Log2FC) ≥ 1 and an adjusted P value (FDR) < 0.05 were identified as differentially expressed genes (DEGs). Volcano plots and heatmaps were generated using the ggplot2 and pheatmap packages in R, respectively, to visualize the expression patterns.

### Molecular docking

We obtained the crystal structures of TNFα, NF-κB1, CASPASE-3, and MAPK3 from the Protein Data Bank (PDB; https://www.rcsb.org/). Next, we downloaded the 3D structure of GAA from PubChem (https://pubchem.ncbi.nlm.nih.gov/). Finally, we performed molecular docking simulations between GAA and each target using AutoDock Vina 1.1.2 and visualized the docking results with Discovery Studio 2019 Client 19.1 software (BIOVIA, San Diego, CA, United States).

### Cell viability assay

RAW264.7 cells were seeded in a 96-well plate at a density of 1 × 10^4 cells per well and incubated at 37 °C with 5% CO_2_. After treatment with GAA at concentrations of 0, 2.5, 5, 10, 20, 40, or 80 μM for 24 h, 10 μL of CCK-8 reagent was added to each well, and absorbance was measured at 450 nm. All procedures were performed according to the manufacturer’s instructions for the Cell Counting Kit-8.

### Flow cytometry

To evaluate macrophage polarization phenotypes, RAW264.7 cells were harvested after the indicated treatments and washed twice with cold PBS. To exclude dead cells from the analysis, cells were first stained with BD Horizon™ Fixable Viability Stain 780 for 15 min at room temperature in the dark. After washing, cells were incubated with an Fragment crystallizable receptor (FcR) blocking solution for 10 min at 4 °C to minimize non-specific binding. For surface marker analysis, cells were stained with PE-Cy7-conjugated anti-CD86 antibody for 30 min at 4 °C. Following surface staining, cells were fixed and permeabilized. Subsequently, cells were stained intracellularly with APC-conjugated anti-CD206 antibody for 30 min. Data acquisition was performed on a flow cytometer (BD biosciences), and data were analyzed using FlowJo v10.8.1 software. The gating strategy involved initial debris exclusion, singlet selection, and dead cell exclusion (FVS780-negative), followed by the quantification of CD86^+^ (M1) and CD206+ (M2) populations (Supplementary Figure S1).

### Molecular dynamics (MD) simulation

GROMACS 2022 was used for all calculations. The protein–ligand complex was constructed using CHARMM36 for the protein and AutoFF-CGenFF for the ligand. It was solvated in a 1 nm TIP3P water box and neutralized with NaCl. Electrostatics were treated using the Particle Mesh Ewald (PME) method with a 1 nm cutoff, and van der Waals interactions were truncated at 1 nm. Hydrogen bonds were constrained using the SHAKE algorithm, and a 1 fs integration time step was employed. A three-stage energy minimization was performed sequentially on water molecules only, ions only, and then the entire all-atom system. This was followed by heating from 0 to 310 K under NVT conditions using the Berendsen thermostat, and a 1 ns NPT equilibration using the Parrinello-Rahman barostat. The production run lasted 100 ns under NPT conditions at 310 K and 1 bar, with coordinates saved every 10 ps. For analysis, the first 10 ns were discarded, and the remaining 90 ns were processed using GROMACS tools to calculate root-mean-square deviation (RMSD), root-mean-square fluctuation (RMSF), hydrogen bond counts, radius of gyration, and solvent-accessible surface area (SASA).

### Surface plasmon resonance (SPR) assay

Surface plasmon resonance (SPR) assay SPR experiments were conducted at 25 °C using a Biacore 8K instrument (Cytiva, MA, United States) equipped with a Series S Sensor Chip CM5. Mouse TNFα protein was immobilized on the active flow cell (Fc2) via standard amine coupling to a target level of approximately 6,000 response units (RU), while the reference flow cell (Fc1) was left unmodified to serve as a control for non-specific binding. The running buffer consisted of 1.05× PBS-P+ containing 5.25% DMSO to match the solvent composition of the analyte samples. To eliminate bulk refractive index variations caused by the high solvent concentration, a solvent correction cycle was performed using eight standard solutions with DMSO concentrations ranging from 4.0% to 5.8%. Ganoderic acid A was serially diluted in the running buffer (ranging from 0.3125 µM to 5 µM) and injected over both flow cells. The dissociation was monitored for 120 s. Data were processed using Biacore Insight Evaluation Software with double referencing (subtraction of the reference surface signal and blank buffer injections) and fitted to a 1:1 Langmuir binding model to determine the kinetic parameters.

### Enzyme-linked immunosorbent assay (ELISA)

Follow the instructions provided with the ELISA kit to measure the expression levels of cytokines (IL-1β, IL-6, IL-10 and TNFα) in mouse serum and cell culture supernatant. Briefly, a sandwich ELISA employing two antibodies was used. The high-affinity enzyme plate was pre-coated with a specific anti-mouse antibody. Samples were added to the wells, and after incubation, the target proteins bound to both the solid-phase and detection antibodies. Following a wash to remove unbound material, streptavidin-HRP was added. After a subsequent wash, the chromogenic substrate TMB was added, and the plate was incubated in the dark to develop color. Finally, absorbance was measured using a microplate reader.

### Quantitative real-time polymerase chain reaction (qRT-PCR) analysis

Under specific conditions, total RNA was extracted from RAW264.7 cells. Reverse transcription was performed using the PrimeScript™ RT reagent kit and qRT-PCR was conducted with the SYBR Green PCR master mix. *β-Actin* served as the internal reference gene. Primer sequences are listed in Supplementary Table S1.

### DIA quantitative proteomics analysis

RAW264.7 cells were treated with vehicle (Control) or TNFα (50 ng/mL) for 12 h, after which they were harvested for proteomic analysis. Total proteins were extracted using SDT lysis buffer and digested with trypsin using the Filter Aided Sample Preparation (FASP) method. The resulting peptide mixtures were analyzed on an Orbitrap Astral mass spectrometer (Thermo Scientific) coupled with a Vanquish Neo UHPLC system in Data-Independent Acquisition (DIA) mode. Raw data were processed using DIA-NN software (version 1.8) against the *Mus musculus* UniProt database. To correct for systematic variation and potential differences in sample loading, Global Normalization was applied to the precursor ion intensities during data processing. Differentially expressed proteins (DEPs) were identified based on a fold change >1.5 and an adjusted P value (FDR) < 0.05 to rigorously control the false discovery rate.

### Western blotting analysis

Mix the tissues and cells with radioimmunoprecipitation assay (RIPA) buffer, add protease and phosphatase inhibitors, sonicate, and incubate on ice. Centrifuge the mixture at 12,000 rpm for 10 min at 4 °C. Then, heat the supernatant at 95 °C for 5 min. Load the tissue or cell lysate onto a 15% sodium dodecyl sulfate–polyacrylamide gel electrophoresis (SDS-PAGE) gel for electrophoresis. Transfer the separated proteins onto a polyvinylidene fluoride (PVDF) membrane. Block the membrane at room temperature with 5% non-fat milk for 1 h, then incubate it at 4 °C with the primary antibody for 10 h. Next, incubate the membrane at room temperature with the secondary antibody for 1 h. Apply the enhanced chemiluminescence (ECL) reagent to the membrane and capture the image using the Tanon 5200 imaging system.

### Immunofluorescence

Animal samples will be fixed with 4% paraformaldehyde, embedded in paraffin, and sectioned. The sections will then undergo dewaxing, cleaning, and antigen retrieval using citrate buffer at high temperature. Subsequently, 5% BSA will be applied for blocking. The sections will be incubated with anti-CD86 (1:200) antibodies at room temperature for 2 h, followed by incubation with a fluorescently labeled secondary antibody for 1 h. After washing with PBS, cell nuclei will be stained with 4′,6-diamidino-2-phenylindole (DAPI), and the samples will be mounted for imaging using a fluorescence microscope.

### Statistical analysis

Data analysis and graph generation were performed using GraphPad Prism 10.1.2. Data are presented as mean+/-standard deviation (SD). The normality of data distribution was assessed using the Shapiro-Wilk test, and homogeneity of variance was verified using the Brown-Forsythe test. For datasets satisfying these assumptions, comparisons between two groups were performed using an unpaired Student’s t-test. For comparisons among multiple groups, one-way analysis of variance (ANOVA) was conducted, followed by Dunnett’s *post hoc* test for comparisons against a control group or Tukey’s *post hoc* test for pairwise comparisons between groups. A *P* value less than 0.05 was considered statistically significant.

## Results

### Network pharmacology and functional enrichment analysis of GAA

**FIGURE 1:**
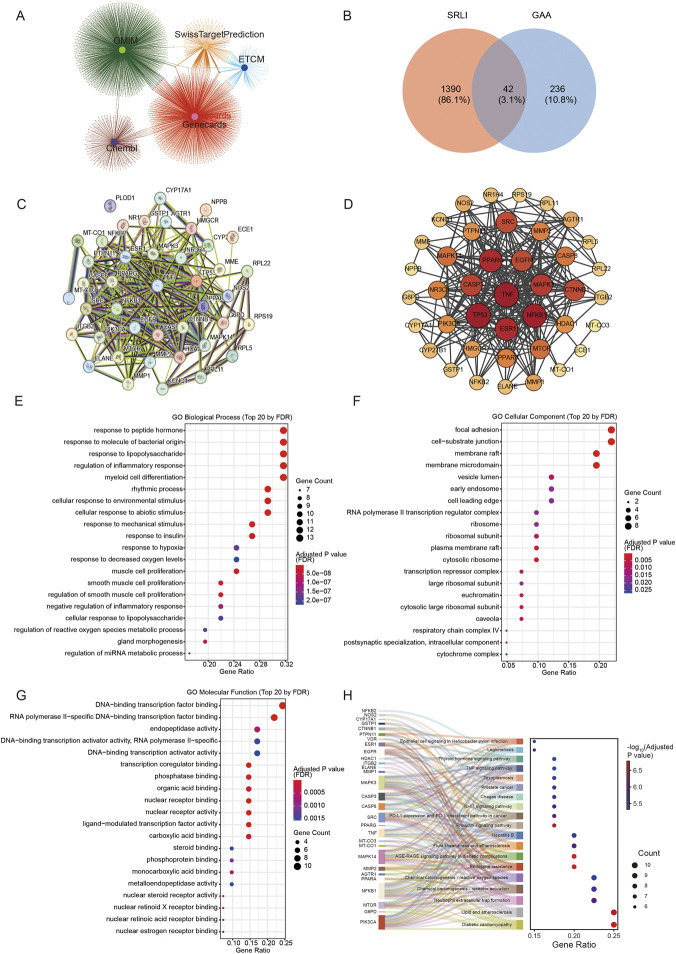
Network pharmacology and functional enrichment analysis of GAA. **(A)** Input the overlapping targets of GAA and SRLI into the EVenn website to construct a Venn network diagram. **(B)** Input the overlapping targets of GAA and SRLI into the EVenn website to generate a Venn diagram. **(C)** Input the overlapping targets of GAA and SRLI into the STRING database to obtain the PPI network. **(D)** Visualize the PPI network using Cytoscape 3.9.1. **(E)** Top 20 BP terms from GO analysis. **(F)** Enriched top20 CC terms from GO analysis. **(G)** Enriched top20 MF terms from GO analysis. **(H)** KEGG-enriched pathways presented as a bubble chart and a Sankey diagram. The Sankey diagram on the left illustrates the relationships between the enriched genes and their corresponding pathways. In all bubble charts, the x-axis represents the gene ratio, and the y-axis indicates the names of the enriched terms. Bubble size corresponds to gene count, and bubble color indicates the adjusted *P* value.

To identify potential targets of GAA in SRLI, we employed a systematic strategy integrating five public databases: ETCM, SwissTargetPrediction, ChEMBL, GeneCards, and OMIM. We retrieved a total of 287 potential targets for GAA and 1432 targets associated with SRLI. A Venn diagram was constructed to illustrate the overlapping targets identified from these databases (Figure 1A), which revealed 42 overlapping targets between GAA and SRLI as candidate therapeutic targets (Figure 1B). Subsequently, we input these 42 candidate targets into the STRING database to construct a PPI network (Figure 1C) and used Cytoscape for further analysis. The core targets were identified based on their degree values (Supplementary Table S2). Eight key targets, including TNF, TP53, NF-κB1, ESR1, CASP3, PPARG, MAPK3, and SRC, were identified as central nodes (Figure 1D). Notably, TNF, NF-κB1, and TP53 exhibited the highest degree values. The prominence of these well-established inflammatory mediators among the hub targets strongly suggests that the therapeutic effect of GAA against SRLI is primarily associated with the modulation of inflammatory responses. Subsequently, Gene Ontology (GO) and Kyoto Encyclopedia of Genes and Genomes (KEGG) analyses of the 42 overlapping targets were performed to elucidate their functional context. In Gene Ontology analysis, terms such as “response to molecule of bacterial origin”, “response to lipopolysaccharide,” and “regulation of inflammatory response” were ranked among the top 20 biological processes (BP) (Figure 1E). Cellular component (CC) analysis highlighted structures like “focal adhesion” and “membrane raft” (Figure 1F). Molecular function (MF) terms were dominated by transcription factor binding and nuclear receptor activity (Figure 1G), indicating that the interaction of GAA with its targets primarily involves signaling transduction and transcriptional regulation. KEGG pathway mapping identified pathways related to inflammation and stress response, including “Lipid and atherosclerosis,” “Chemical carcinogenesis - receptor activation,” and notably, the “TNF signaling pathway” and “IL-17 signaling pathway” (Figure 1H). Collectively, these computational results highlight the TNF signaling pathway as a key potential node for GAA’s intervention in SRLI, forming the central hypothesis for our subsequent experimental validation.

### Validation of core targets in an SRLI model

**FIGURE 2:**
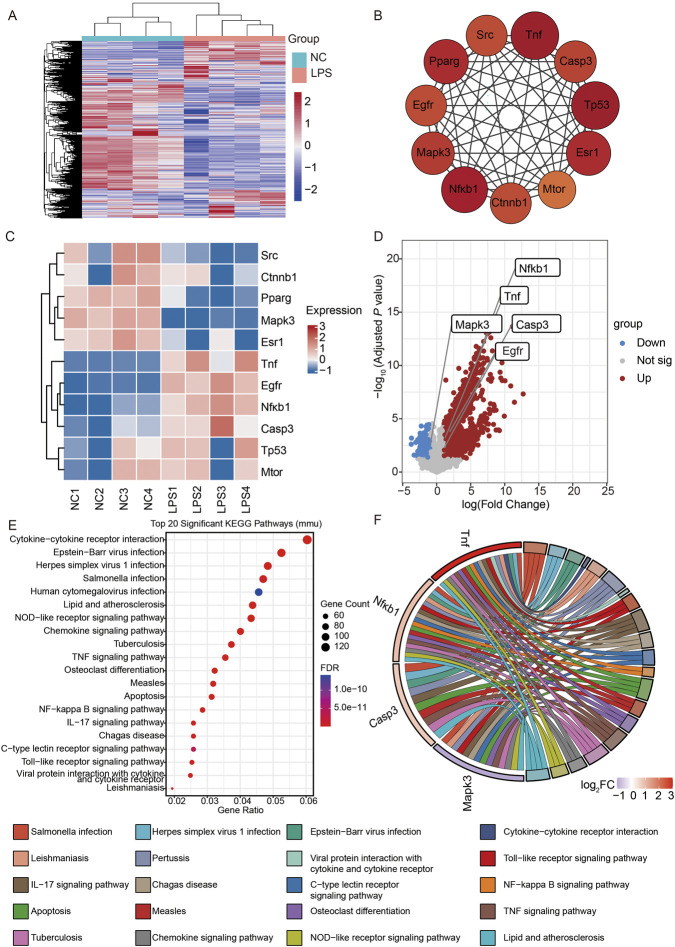
Validation of core targets in an SRLI model (GSE217695). **(A)** Heatmap of liver gene expression levels in wild-type and SRLI mice (n = 4). **(B)** Construction of PPI network for key targets using Cytoscape. **(C)** Heatmap of expression levels of 11 key target genes in the livers of wild-type and SRLI mice. **(D)** Volcano plot showing the expression levels of differentially expressed genes (DEGs). **(E)** KEGG enrichment analysis of liver DEGs (WT vs. LPS-induced SRLI). **(F)** KEGG chord plot illustrating core targets and their associated injury-related pathways in SRLI liver.

To independently validate the hub genes identified through network pharmacology, we reanalyzed the publicly available murine hepatic transcriptome dataset GSE217695. Eight hours after LPS challenge, differential expression analysis revealed a clear distinction between control and septic livers (Figure 2A). From the PPI network, we identified 11 high-degree nodes (degree ≥18) as putative drivers of the LPS-induced signature (Figure 2B). Expression profiling of these candidates showed significant upregulation of Tnf, *Nf-κb1*, Casp3, and Egfr, along with downregulation of Mapk3, while Pparg, Esr1, Tp53, Mtor, Ctnnb1, and Src remained unchanged (Figure 2C). The significant upregulation of Tnf, *Nf-κb1*, Casp3, and Egfr provided strong independent validation for our network pharmacology predictions, and these four genes, together with the downregulated Mapk3, were therefore selected as the core set for further investigation (Figure 2D). Collectively, the dysregulation of these five genes underscores their central role in the pathogenesis of SRLI and, in conjunction with our network pharmacology results, strongly nominates them as high-priority candidate targets for GAA. KEGG enrichment analysis of the 8-h LPS-induced hepatic transcriptome identified “Cytokine-cytokine receptor interaction” as the top significant pathway. Additionally, inflammation-related pathways such as “TNF signaling,” “NF-kappa B signaling,” and “IL-17 signaling” were significantly enriched, along with “*Salmonella* infection”-related immune cascade (Figure 2E). A Sankey plot (Figure 2F) further illustrated the flow of DEGs into these pathways, positioning Tnf and Nfκb1 as central nodes propagating the inflammatory signal. These findings align with the GO terms related to immune and migration processes (Supplementary Figure S2) and reinforce that hyperactivation of the TNFα/NF-κB axis is a principal transcriptional hallmark of SRLI, thereby providing a strong rationale and a focused direction for evaluating GAA intervention.

### Molecular docking of primary targets of GAA

**FIGURE 3:**
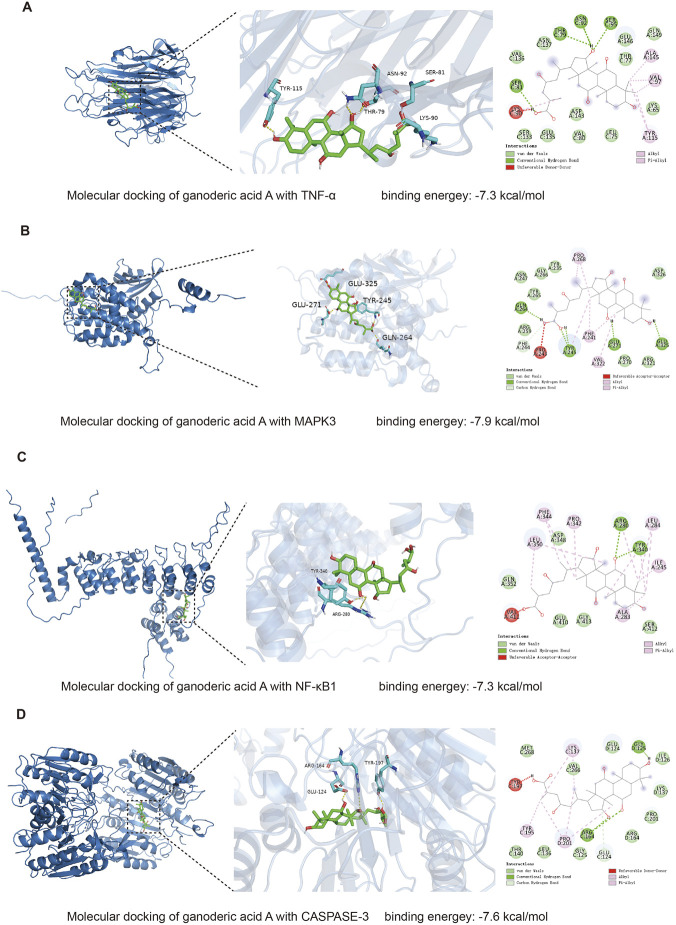
Molecular Docking of Primary Targets of GAA. **(A)** Molecular docking visualization of TNFα with GAA. Binding energy: 7.3 kcal/mol. **(B)** Molecular docking visualization of NF-κB1 with GAA. Binding energy: 7.3 kcal/mol. **(C)** Molecular docking visualization of MAPK3 with GAA. Binding energy: 7.9 kcal/mol. **(D)** Molecular docking visualization of CASPASE-3 with GAA. Binding energy: 7.6 kcal/mol.

Convergent evidence from network pharmacology and transcriptomics identified several central hubs, including TNFα, NF-κB1, MAPK3, and CASP3. The selection of TNFα for subsequent experimental validation was driven by a convergent multi-layered analysis: it emerged as a top-ranked hub node in both our network pharmacology and independent transcriptomic PPI networks, and its position as a master upstream regulator of the NF-κB pathway—the most significantly enriched pathway in our KEGG analysis—suggested that its direct targeting could mechanistically explain the suppression of the entire inflammatory cascade. Molecular docking revealed that all four proteins displayed strong affinities for GAA, with binding energy ≤ −7.3 kcal/mol (MAPK3 = −7.9 kcal/mol, CASP3 = −7.6 kcal/mol, TNFα = −7.3 kcal/mol, NF-κB1 = −7.3 kcal/mol) (Figures 3A–D). Inspection of the TNFα–GAA complex showed the triterpenoid docked into the cytokine’s active site, forming four direct hydrogen bonds (Ser95, Asn92, Thr79, Ser81) that provide enthalpic stability through key molecular interactions (Supplementary Table S3).

### Biophysical validation of the GAA-TNFα interaction via SPR and molecular dynamics simulation

**FIGURE 4:**
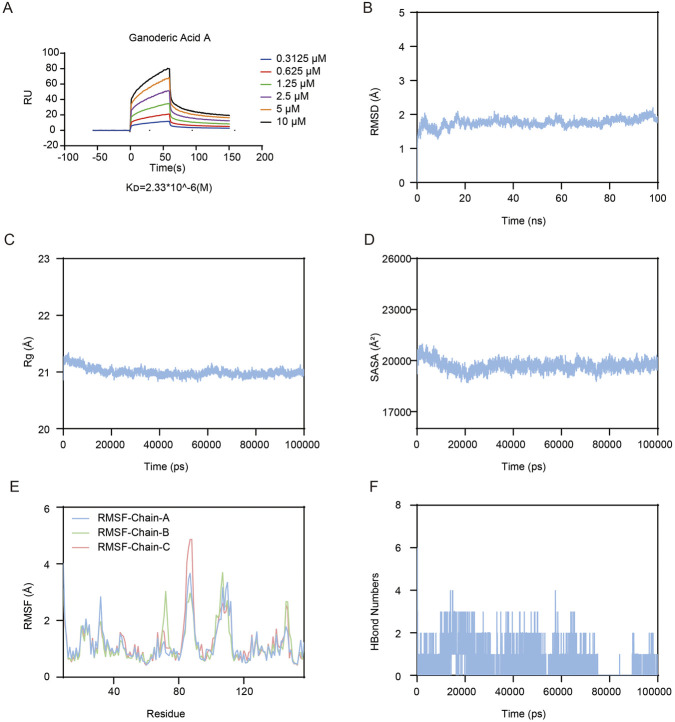
Biophysical Validation of the GAA-TNFα Interaction via SPR and Molecular Dynamics Simulation. **(A)** SPR analysis conducted under the indicated conditions to assess the binding between GAA and TNFα. **(B)** RMSD of the TNFα/GAA complex. **(C)** Rg of the TNFα/GAA complex. **(D)** SASA of the TNFα/GAA complex. **(E)** RMSF of the TNFα/GAA complex. **(F)** Hydrogen bonding analysis of the TNFα/GAA complex.

To confirm the physical interaction, we measured GAA binding to TNFα by surface plasmon resonance (SPR). Fitting the sensorgrams to a 1:1 binding model yielded an equilibrium dissociation constant (K_D_) of 2.3 µM (Figure 4A), an affinity typical for small-molecule cytokine inhibitors. We then performed a 100-ns molecular dynamics (MD) simulation to assess the stability of the complex. The root-mean-square deviation (RMSD) plateaued within 20 ns and remained stable below 3 Å (Figure 4B), indicating a well-equilibrated system. Simultaneous decreases in solvent-accessible surface area (SASA) and radius of gyration (Rg) (Figures 4C,D) indicated that GAA binding induces a more compact conformation in TNFα. This ligand-induced structural tightening may potentially interfere with the conformational dynamics required for TNFα trimerization or receptor binding. At the residue level, the root-mean-square fluctuation (RMSF) remained low (>95% of residues <3 Å) (Figure 4E), while one to three persistent hydrogen bonds maintained the ligand’s position throughout the simulation (Figure 4F). In summary, the micromolar affinity from SPR and the multi-parameter stability profile from MD confirm that GAA directly binds to TNFα. Our prior bioinformatic analyses (Figures 1H, 2E,F) consistently positioned the NF-κB signaling pathway as the most significantly enriched cascade downstream of TNFα in SRLI, and PPI networks highlighted NF-κB1 as a core hub. We therefore hypothesize that the primary mechanism by which GAA-TNFα binding exerts its anti-inflammatory effect is through the suppression of this dominant NF-κB axis. This central hypothesis was tested and validated in our subsequent cellular experiments.

### GAA inhibits TNFα-driven inflammation in macrophages by suppressing NF-κB signaling

**FIGURE 5:**
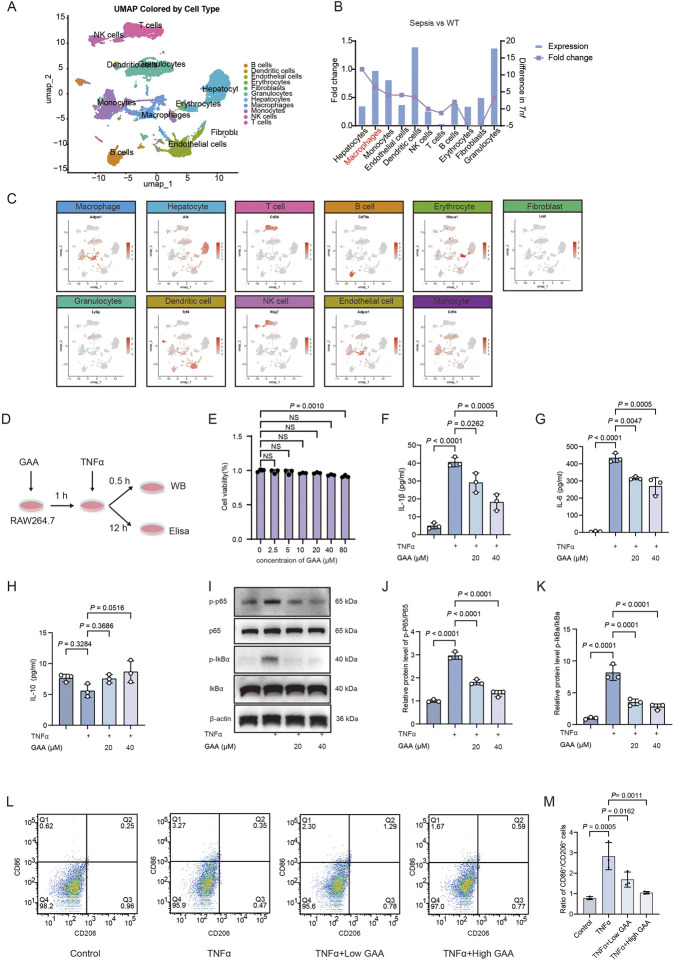
GAA inhibits TNFα-driven inflammation in macrophages by suppressing NF-κB signaling. **(A)** UMAP plot of scRNA-seq data (GSE279167) from septic mouse liver tissues, colored by annotated cell types. **(B)** The fold change (left) and basal expression level (right) of Tnf expression in major cell types between septic mice and wild-type mice liver. **(C)** Expression levels of known marker genes in unclassified cells from liver tissue, displayed on a UMAP plot. **(D)** Schematic of the *in vitro* experimental protocol: RAW264.7 macrophages were pretreated with GAA prior to TNFα stimulation, followed by Western blot and ELISA analysis. **(E)** Viability of RAW264.7 cells treated with increasing concentrations of GAA (0–80 μM) for 24 h, assessed by CCK-8 assay. **(F–H)** ELISA measurements of **(F)** IL-1β, **(G)** IL-6, and **(H)** IL-10 in culture supernatants of RAW264.7 cells pretreated with GAA (20, 40 μM) and stimulated with TNFα. **(I–K)** Representative Western blots and statistics of p65, phosphorylated p65 (p-p65), and IκBα in cell lysates. **(L)** Flow cytometric analysis of M1 and M2 macrophage polarization. **(M)** Bar chart shows the quantitative analysis results of the ratio of CD86^+^ cells to CD206^+^ cells. Data are presented as mean ± SD of n = 3 independent biological replicates. Statistical significance was determined by one-way ANOVA followed by Tukey’s *post hoc* test. *P* values are indicated directly in the figures.

To identify the primary cellular effector of TNFα in septic liver injury for downstream validation, we analyzed a public scRNA-seq dataset. Tnf expression was significantly upregulated across cell populations (Figures 5A,B). A critical comparative analysis revealed that although hepatocytes showed the highest fold-change, this was attributable to their exceptionally low baseline expression. In contrast, macrophages not only maintained a substantially higher baseline level of Tnf but also exhibited a pronounced fold-increase upon insult, establishing them as the dominant and most relevant source of pathogenic TNFα signaling in the septic liver microenvironment (Figure 5C). This finding rationally guided our selection of the RAW264.7 macrophage model for functional validation. Cell viability assays confirmed no significant cytotoxicity of GAA at the tested concentrations (20 and 40 µM), ensuring that subsequent functional interpretations were not confounded by reduced cell viability (Figure 5E). We next established a model of TNFα challenge using recombinant cytokine (rTNFα) to test GAA’s functional antagonism. To unbiasedly identify the key pathways engaged by TNFα in this model, we first performed proteomic analysis of rTNFα-stimulated macrophages. KEGG enrichment analysis of the differentially expressed proteins identified the NF-κB signaling pathway as the most significantly enriched cascade (Supplementary Figure S3), providing a data-driven rationale for our subsequent mechanistic focus. Stimulation with rTNFα robustly triggered the secretion of the pro-inflammatory cytokines IL-1β and IL-6 (Figures 5F,G). Pre-treatment with GAA potently suppressed this release in a dose-dependent manner. In contrast to some anti-inflammatory agents, GAA did not significantly alter the secretion of IL-10 under these conditions (Figure 5H), indicating that its primary action is the suppression of pro-inflammatory signaling. Guided by the proteomic results, we investigated the NF-κB pathway. Western blot analysis confirmed that rTNFα stimulation for 30 min induced rapid phosphorylation of the p65 subunit and its inhibitor IκBα (Figures 5I–K), marking pathway activation. GAA pre-treatment effectively blunted this early signaling event, demonstrated by a dose-dependent reduction in the levels of both p-p65 and p-IκBα. This result provides direct evidence that GAA intercepts TNFα signaling at the level of the early NF-κB activation cascade. Furthermore, flow cytometric analysis revealed that GAA pre-treatment dose-dependently decreased M1/M2 ratio (Figures 5L,M), indicating a functional restoration of a balanced phenotypic state. Collectively, our data demonstrate that GAA functions as a potent antagonist of TNFα-induced pro-inflammatory signaling in macrophages. It effectively blocks the early activation of the NF-κB pathway, leading to reduced synthesis of key pro-inflammatory cytokines and a concomitant decrease in M1 polarization.

### GAA Ameliorates septic liver injury by targeting the TNFα/NF-κB axis

**FIGURE 6:**
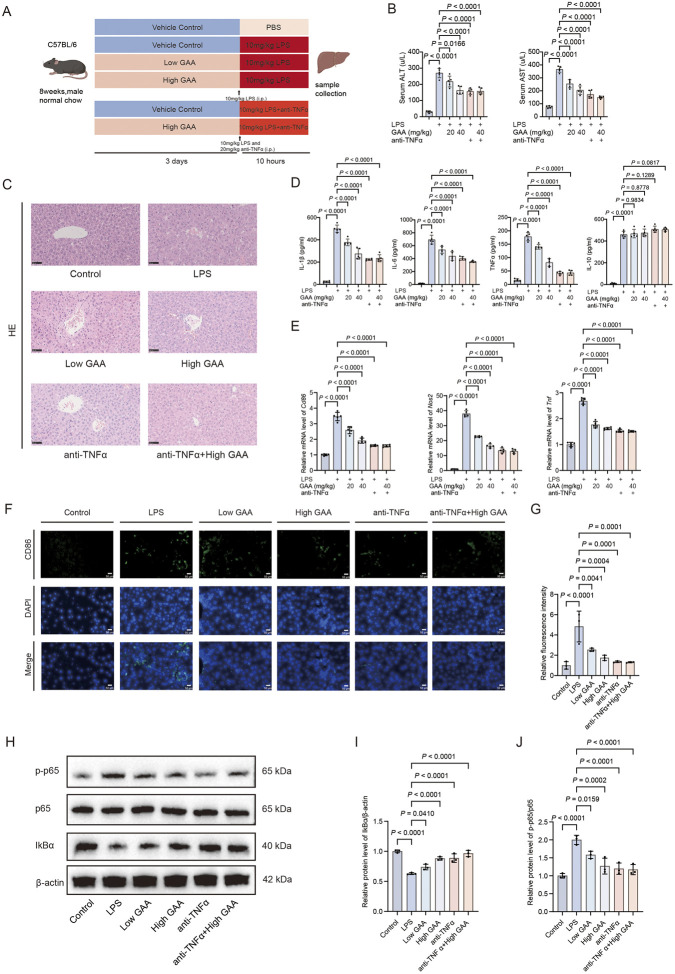
GAA Ameliorates Septic Liver Injury by Targeting the TNFα/NF-κB Axis. **(A)** Schematic of the experimental timeline. C57BL/6 male mice were randomly assigned to six groups: (1) Vehicle control; (2) LPS; (3) LPS + Low GAA (20 mg/kg); (4) LPS + High GAA (40 mg/kg); (5) LPS + anti–TNFα antibody; (6) LPS + anti–TNFα antibody + High GAA (40 mg/kg). GAA or its vehicle was administered orally for 3 days prior to LPS challenge. **(B)** Serum levels of alanine aminotransferase (ALT) and aspartate aminotransferase (AST) (n = 5 mice per group). **(C)** Representative hematoxylin and eosin **(H,E)**-stained liver sections showing the periportal areas from different groups of mice. Scale bar, 50 µm. **(D)** Serum concentrations of IL-1β, IL-6, TNFα, and IL-10 measured by ELISA (n = 5 mice per group). **(E)** Hepatic mRNA levels of *Cd86, Nos2,* and *Tnf* determined by qPCR (n = 5 mice per group). **(F)** Representative immunofluorescence images of liver sections stained for the M1 macrophage marker CD86 (green) and DAPI (blue). Scale bar, 50 µm. **(G)** Quantitative analysis of CD86 fluorescence intensity (n = 3 biological replicates). **(H)** Representative Western blots of phosphorylated p65 (p-p65), total p65, and IκBα in liver lysates. **(I,J)** Densitometric quantification of the p-p65/p65 ratio and IκBα protein level. Data are presented as mean ± SD (n = 3 biological replicates). Statistical significance was determined by one-way ANOVA followed by Tukey’s *post hoc* test. *P* values are indicated directly in the figures.

To validate the therapeutic efficacy and target specificity of GAA *in vivo*, we established an LPS-induced sepsis-associated liver injury model in mice. The experimental design included six groups: Control, LPS, LPS + Low GAA, LPS + High GAA, LPS + anti-TNFα (positive control), and LPS + anti-TNFα + High GAA (Figure 6A). Administration of GAA markedly improved LPS-induced liver injury, as evidenced by a significant reduction in serum alanine aminotransferase (ALT) and aspartate aminotransferase (AST) levels (Figure 6B) and a clear amelioration of histopathological lesions, including disrupted hepatic cord structure and focal necrosis (Figure 6C). The hepatoprotective effect of the High GAA dose was comparable to that of the anti-TNFα antibody, with no significant difference observed between the two groups. At the molecular level, Western blot analysis of liver lysates (Figure 6H) revealed that LPS challenge significantly enhanced p65 phosphorylation (p-p65) and promoted IκBα degradation, which were significantly reversed by GAA treatment, substantiating its inhibition of the NF-κB pathway *in vivo*. Crucially, the combination of High GAA with anti-TNFα antibody yielded no significant additive inhibitory effects on NF-κB pathway activation compared to anti-TNFα treatment alone (Figures 6H–J). Consistent with the suppression of systemic inflammation, the LPS-triggered surge in serum pro-inflammatory cytokines (IL-1β, IL-6, TNFα) was significantly suppressed by GAA (Figure 6D). Furthermore, GAA significantly reduced the LPS-induced upregulation of inflammatory genes (*Cd86, Nos2, Tnf*) (Figure 6E) and inhibited pro-inflammatory macrophage polarization, as evidenced by decreased CD86^+^ immunofluorescence in liver sections (Figures 6F,G). In summary, GAA demonstrates potent hepatoprotective and anti-inflammatory effects in a murine model of SRLI, mechanistically linked to the suppression of the NF-κB pathway. The powerful non-additivity observed upon co-treatment with an anti-TNFα antibody demonstrates that the therapeutic mechanisms of GAA and anti-TNFα antibody are not independent and likely share a critical, rate-limiting node within the TNFα signaling pathway.

## Discussion

The hepatoprotective effects of the medicinal mushroom *G. lucidum* have been widely documented, with GAA identified as one of its principal bioactive triterpenoids (Chen et al., 2023; Chen et al., 2025; Zhao et al., 2024). However, the precise molecular mechanism by which GAA confers protection against SRLI remains inadequately defined. This study aimed to elucidate this mechanism by systematically investigating its potential interaction with the TNFα signaling axis. Our integrated approach, combining computational prediction, biophysical analysis, and functional validation, provides compelling evidence that GAA alleviates SRLI primarily by functioning as a potent antagonist of TNFα-driven inflammation, thereby disrupting the NF-κB signaling cascade and subsequent pro-inflammatory macrophage activation.

TNFα orchestrates multiple inflammatory events, from cytokine release to the initiation of cell-death cascades (Burger et al., 2023; Jang et al., 2021). During the acute phase of septic shock its influence is particularly pronounced, serving as a principal mediator (Wu et al., 2024). Our findings position TNFα as a highly plausible central target for GAA. The initial prediction from network pharmacology, which identified TNFα as a key node, was consistently supported by downstream experimental data. However, it is important to note that our molecular docking analysis also revealed strong binding affinities of GAA for other targets, specifically MAPK3 (−7.9 kcal/mol) and CASP3 (−7.6 kcal/mol). This suggests that GAA may possess multi-target pharmacology characteristic of natural products. Although our biophysical and functional data prioritize the TNFα blockade as a critical upstream event, we cannot rule out that GAA may also directly modulate MAPK3 or downstream apoptotic effectors, thereby exerting a synergistic anti-inflammatory effect. While molecular docking suggested a stable binding mode, and surface plasmon resonance (SPR) quantified this interaction with micromolar affinity (K_D_ = 2.3 µM), we acknowledge that these techniques alone cannot conclusively prove direct functional targeting in a cellular context. They do, however, provide a strong foundational hypothesis. The most persuasive functional evidence for GAA’s engagement with the TNFα pathway comes from our *in vivo* combination therapy experiment. The absence of any additive therapeutic effect when GAA was co-administered with a saturating dose of an anti-TNFα antibody strongly suggests that the protective mechanisms of both agents are not independent and likely converge on the same critical pathway.

The functional consequence of this TNFα pathway engagement is the effective activation of the downstream NF-κB signaling cascade (Bakshi et al., 2022). Our data robustly demonstrate that GAA treatment blunts TNFα-induced phosphorylation of p65 and prevents the degradation of IκBα in both cellular and animal models. This interception of a central inflammatory signaling axis provides a coherent explanation for the observed attenuation of systemic and hepatic inflammation, including the reduction in key pro-inflammatory cytokines (IL-1β, IL-6).

A critical downstream effect of this pathway suppression is the effective restraint of pro-inflammatory macrophage polarization (Arabpour et al., 2021; Wang and Wang, 2023). In SRLI, overabundant M1 macrophages is a key driver of liver injury (Hou et al., 2025; Zhou et al., 2023). Our findings clearly indicate that GAA significantly suppresses the M1 polarization program, as evidenced by the downregulation of canonical M1 markers (CD86, NOS2) and a reduction in CD86^+^ macrophage populations in injured liver tissue. It is noteworthy that GAA treatment did not significantly enhance the secretion of the anti-inflammatory cytokine IL-10. This observation suggests that the primary anti-inflammatory action of GAA is rooted in the suppression of the pro-inflammatory arm, rather than the active promotion of alternative anti-inflammatory pathways. By potently inhibiting the dominant M1-driver signal (the TNFα/NF-κB axis), GAA effectively halts the progression of inflammation, with the dampening of the M1 phenotype representing a key component of this therapeutic effect.

Beyond simply blocking the extracellular TNFα signal, our findings hint at a more profound mechanism through which GAA may exert its sustained anti-inflammatory effect. We observed that GAA treatment significantly suppressed the transcription of the Tnf gene itself in the septic liver. Given that NF-κB is a master transcriptional regulator of Tnf gene expression, we propose that GAA, by inhibiting the TNFα-NF-κB axis, disrupts a key positive feedback loop that normally amplifies and perpetuates inflammation. In this model, the initial inhibition of TNFα signaling reduces NF-κB activation, which in turn leads to decreased *de novo* synthesis of TNFα, thereby preventing a self-reinforcing inflammatory cascade. While further research is needed to fully validate this circuit, our data position GAA as an agent capable of interrupting this vicious cycle.

Despite this multi-faceted evidence, our study has limitations that delineate clear avenues for future research. Foremost is the need for direct structural and functional validation of the GAA-TNFα interaction. Techniques such as X-ray co-crystallography and cellular assays like TNFα-TNFR binding ELISAs are required to unequivocally confirm direct targeting and functional antagonism. Furthermore, the potential for GAA to cause systemic immunosuppression at effective anti-septic doses remains unexplored. Additionally, while the LPS-induced acute liver injury model is widely used, it primarily mimics the early hyper-inflammatory phase of endotoxemia rather than the complex, dynamic hemodynamic and immunological alterations seen in full clinical sepsis. Future studies utilizing cecal ligation and puncture (CLP) models would be beneficial to validate these findings in a more clinically relevant context. These experiments will be further refined and conducted in future studies.

In conclusion, our work provides a mechanistically grounded model for the action of GAA in SRLI. We propose that GAA acts as a potential TNFα pathway modulator, which leads to the suppression of NF-κB signaling and a resultant suppression of pro-inflammatory M1 macrophage polarization. While further validation is needed to cement the direct nature of the interaction, this target-centric understanding significantly advances the pharmacological characterization of a traditional remedy and positions GAA as a promising lead compound for targeted immunomodulation in SRLI.

## Conclusion

In summary, our study elucidates a novel mechanism by which GAA protects against sepsis-related liver injury. By identifying TNFα as a direct physical target, we demonstrate that GAA functions as a natural small-molecule inhibitor that disrupts the upstream inflammatory cascade. This blockade effectively suppresses the canonical NF-κB pathway and downstream M1 macrophage polarization, breaking the vicious cycle of hepatic inflammation. These findings not only advance the pharmacological characterization of Ganoderma lucidum but also highlight the therapeutic promise of targeting the TNFα/NF-κB axis with natural products for managing severe liver pathologies.

## Data Availability

The datasets presented in this study can be found in online repositories. The names of the repository/repositories and accession number(s) can be found in the article/Supplementary Material.
