# Supplementary material for: Multi-omics integration identifies ganoderic acid A as a TNFα inhibitor for treating sepsis-related liver injury
Source: Front Pharmacol. 2026 Jan 26;17:1754172. doi: 10.3389/fphar.2026.1754172 (PMC12884166; doi:10.3389/fphar.2026.1754172)
Supplement: Supplementary file 1 [file Supplementaryfile1.docx]

# Table S1. Primer sequences use in this research.

| **Gene** | **Direction** | **Base sequence** |
| --- | --- | --- |
| *Tnf* | Forward | 5‘-ACGGCATGGATCTCAAAGAC-3’ |
|  | Reverse | 5‘-GTGGGTGAGGAGCACGTAGT-3’ |
| *Cd86* | Forward | 5‘-TCAATGGGACTGCATATCTGCC-3’ |
|  | Reverse | 5‘-GCCAAAATACTACCAGCTCACT-3’ |
| *Nos2* | Forward | 5‘-GTTCTCAGCCCAACAATACAAGA-3’ |
|  | Reverse | 5‘-GTGGACGGGTCGATGTCAC-3’ |
| *Actin* | Forward | 5‘-GTGACGTTGACATCCGTAAAGA-3’ |
|  | Reverse | 5‘-GCCGGACTCATCGTACTCC-3’ |

# Table S2. The 9 most significant targets of PPI network.

| Number | Unipro ID | Target name | Degree |
| --- | --- | --- | --- |
| 1 | P01375 | TNF | 30 |
| 2 | P04637 | TP53 | 30 |
| 3 | P19838 | NF-κB1 | 28 |
| 4 | P03372 | ESR1 | 26 |
| 5 | P37231 | PPARG | 26 |
| 6 | P27361 | MAPK3 | 24 |
| 7 | P42574 | CASP3 | 23 |
| 8 | P35222 | CTNNB1 | 22 |
| 9 | P12931 | SRC | 22 |

# Table S3. Hydrogen-bond interactions between ganoderic acid A and selected target proteins.

| Drug | Targets | PDB ID | Binging Energy (kJ/mol) | Hydrogen Bonds |
| --- | --- | --- | --- | --- |
| Ganoderic acid A | CASPASE-3 | 3DEI | −7.6 | ARG164、GLY125 |
|  | TNF-α | 1TNF | −7.3 | SER95、ASN92、THR79、SER81 |
|  | NF-κB1 | 1NFK | −7.3 | ARG280、TYR340 |
|  | MAPK3 | 4QTB | −7.9 | GLN264、TYR245、GLU271、GLU325 |

# Table S4. Reagents and chemicals

| **Reagent or resource** | **Identifier** | **Source** |
| --- | --- | --- |
| **Antibodies** | | |
| NF-κB p65 | 8242 | Cell Signaling Technology |
| phospho-NF-κB p65 | 3033 | Cell Signaling Technology |
| IκBα | 4814, | Cell Signaling Technology |
| phospho-IκBα | 2859 | Cell Signaling Technology |
| Anti-rabbit IgG Antibody | 7074 | Cell Signaling Technology |
| Anti-mouse IgG Antibody | 7076 | Cell Signaling Technology |
| β-actin | 20536 | Proteintech Biotech co.,ltd |
| CD206 | S0B5331-100T | STARTER |
| CD86 | 560582 | BD Pharmingen |
| BD Horizon™ Fixable Viability Stain 780 | 565388 | BD Pharmingen |
| CD86 | 13395-1-AP | Proteintech |
| **Chemicals** | | |
| Ganoderic acid A (99% purity) | HY-N1447 | MedChemExpress |
| Lipopolysaccharide (LPS) (Escherichia coli serotype) | 0111: B4 | Sigma-Aldrich |
| Recombinant Mouse TNF-alpha Protein | RP01071 | Abclonal |
| SYBR Green PCR master mix | G3326 | Service Biotech co.,ltd |
| HiScript III All-in-one RT SuperMix | R333-01 | Vazyme Biotech co.,ltd |
| Anti-Mouse TNF-alpha Antibody | HY-P990794 | MedChemExpress |
| Fixation/Permeabilization Kit | Abs9936 | Absin |
| Mouse FcR Blocking Reagent | S0B0599 | STARTER |
| DAPI | abs47047616 | Absin |
| **Critical commercial assays** |  |  |
| BCA protein assay kit | ZJ102 | Yamay Biotech co.,ltd |
| Dulbecco’s modified Eagle’s medium | G4511PS | Service Biotech co.,ltd |
| RMPI-1640 medium | G4531PS | Service Biotech co.,ltd |
| Fetal bovine serum, Australia | A3160801 | Gibco |
| Cell Counting Kit-8 | C0037 | Beyotime |
| Mouse IL-1β ELISA Kit | 88-7013 | Invitrogen |
| Mouse TNF-a ELISA Kit | 88-7324 | Invitrogen |
| Mouse IL-10 ELISA Kit | 88-7105 | Invitrogen |
| Mouse IL-6 ELISA Kit | 88-7064 | Invitrogen |

# Supplementary Figure 1


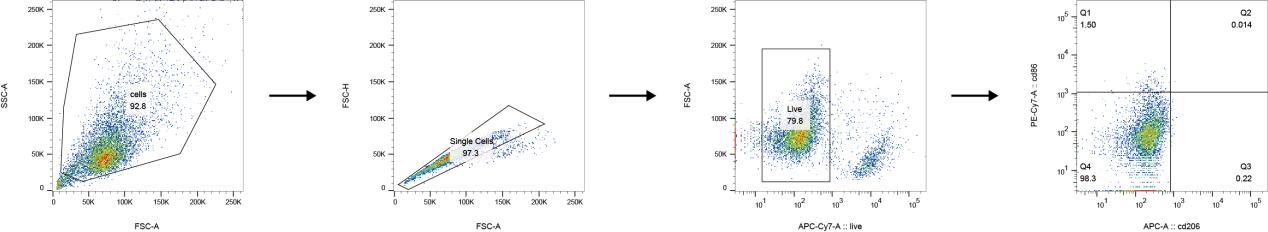


Figure S1. Gating strategy of flow cytometry.

# Supplementary Figure 2


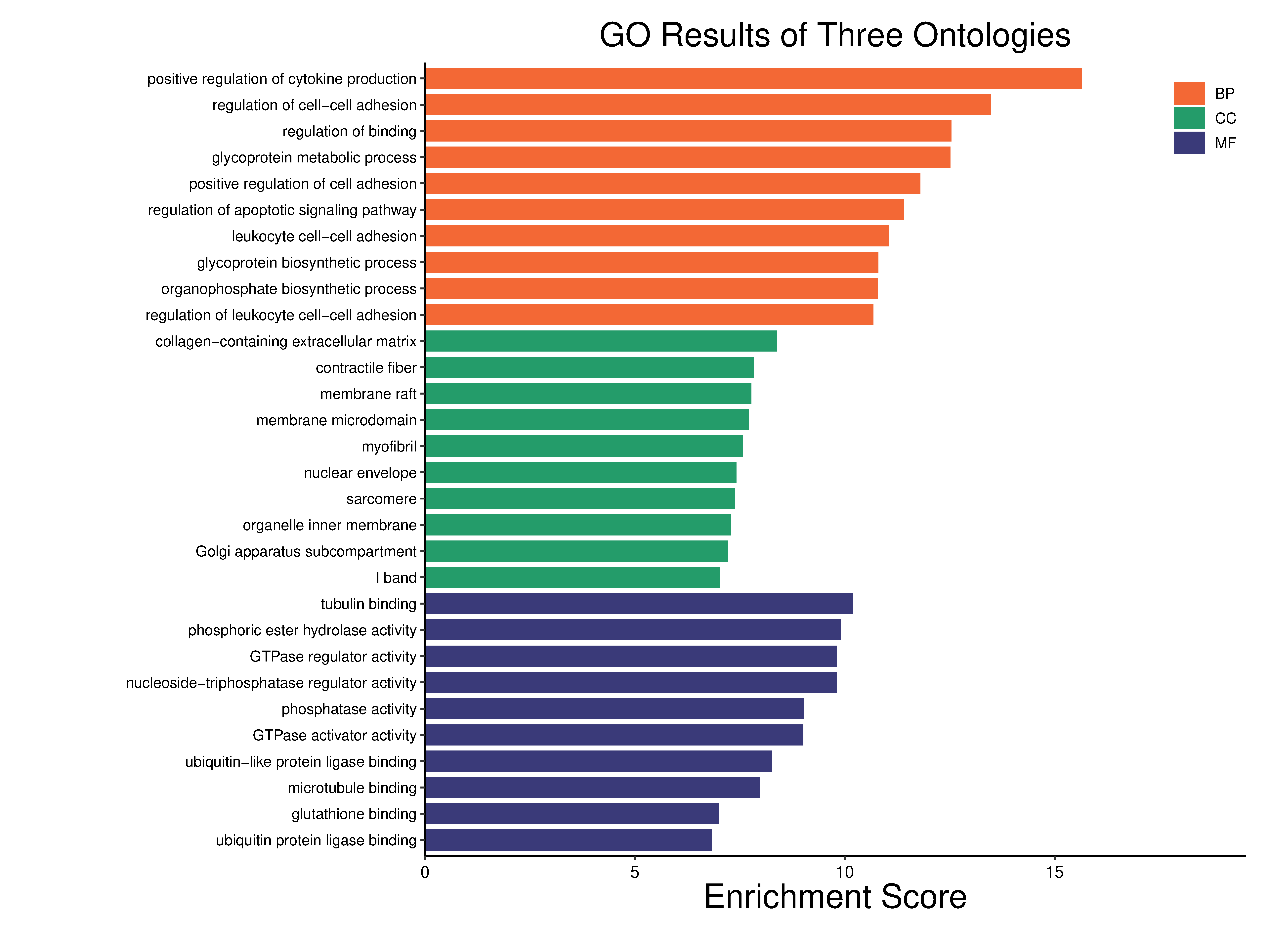


Figure S2. GO analysis enrichment of differentially expressed genes (DEGs) in liver (SRLI vs WT).

# Supplementary Figure 3
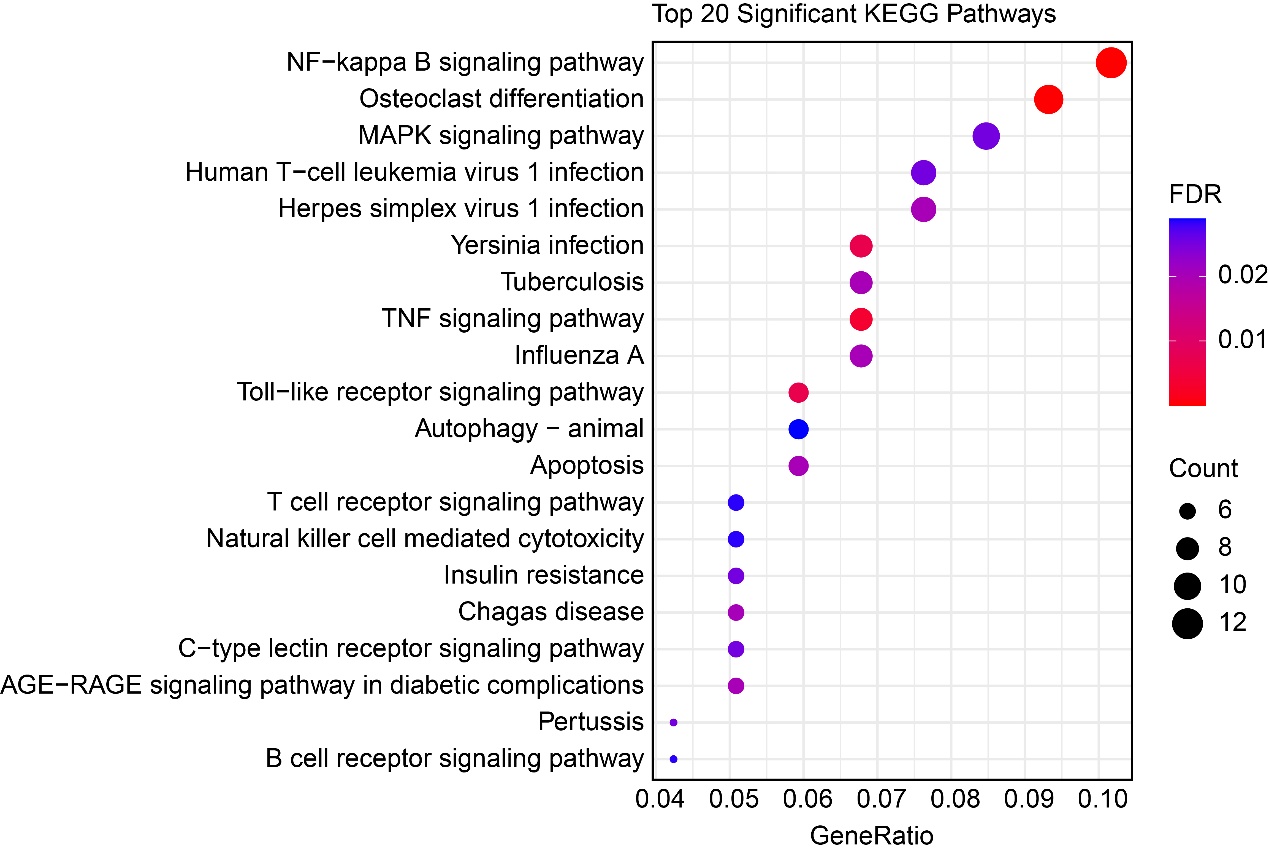


Figure S3. KEGG enrichment analysis of differentially expressed proteins (DEPs) in RAW264.7 macrophages stimulated with TNFα (TNFα-challenged vs Control).

# Supplementary Figure 4
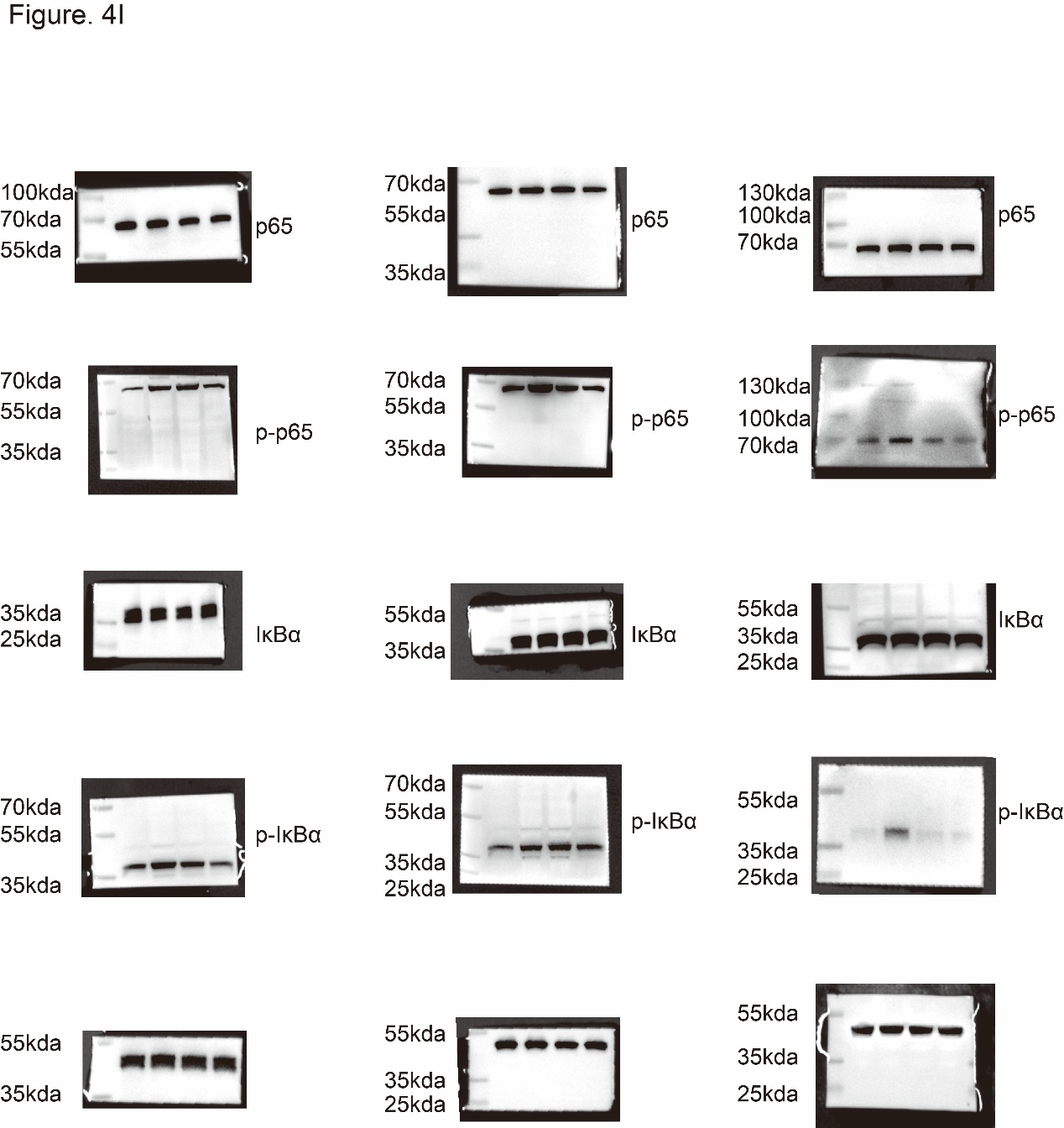


Supplementary Figure 4. Uncropped Western blot images corresponding to Figure 4I. The original, uncropped scans of the Western blots for p65, p-p65, IκBα, and p-IκBα are presented. Molecular weight markers are indicated on the left of each band.

**Supplementary Figure 4**
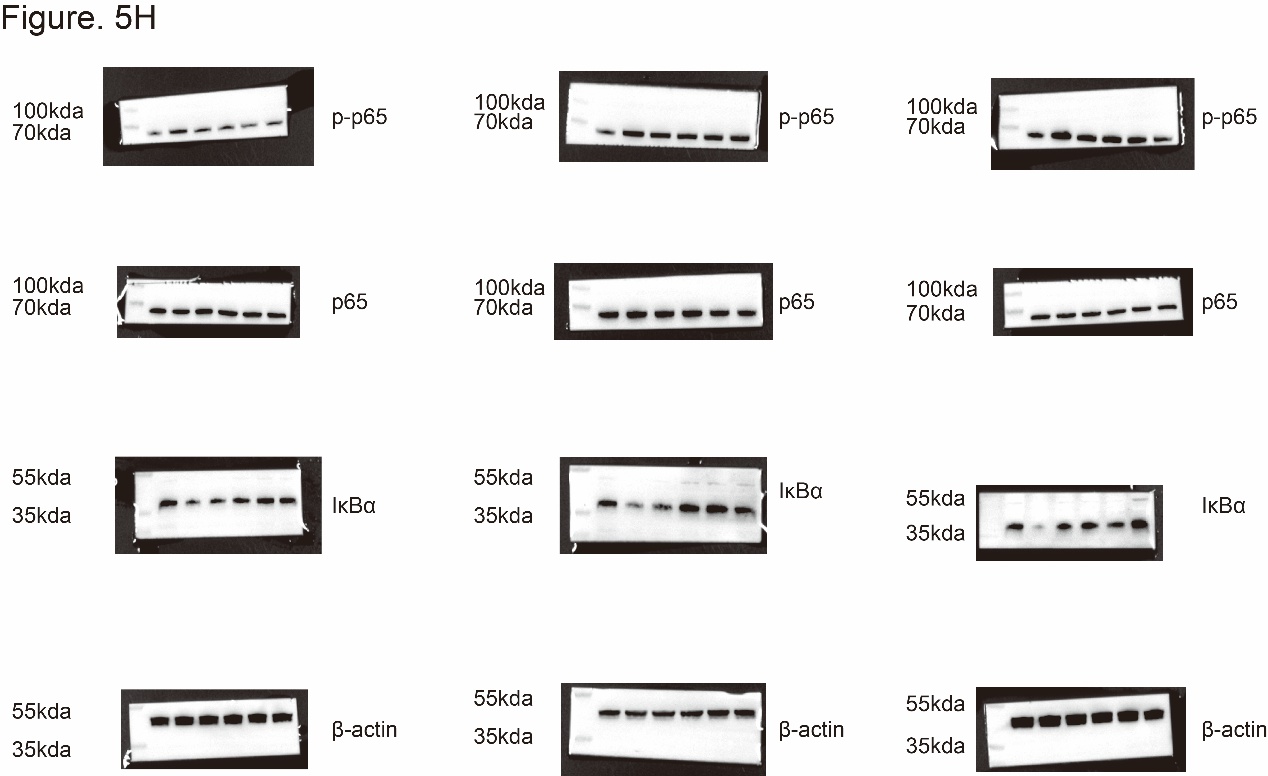


Supplementary Figure 5. Uncropped Western blot images corresponding to Figure 5H. The original, uncropped scans of the Western blots for p-p65, p65, IκBα, and β-actin are presented. Molecular weight markers are indicated on the left.
